# Swine Influenza Virus Infection Decreases the Protective Immune Responses of Subunit Vaccine Against Porcine Circovirus Type 2

**DOI:** 10.3389/fmicb.2021.807458

**Published:** 2021-12-24

**Authors:** Yuhang Sun, Jinlong Zhang, Zixuan Liu, Ying Zhang, Kehe Huang

**Affiliations:** ^1^Key Laboratory of Zoonosis of Liaoning Province, College of Animal Science and Veterinary Medicine, Shenyang Agricultural University, Shenyang, China; ^2^Department of Animal Nutrition and Immunology, College of Veterinary Medicine, Nanjing Agricultural University, Nanjing, China

**Keywords:** swine influence virus, subunit vaccine, inactivated vaccine, protectively immune responses, macrophage polarization, porcine circovirus type 2

## Abstract

Porcine circovirus type 2 (PCV2) is the primary pathogen of porcine circovirus diseases and porcine circovirus associated diseases. Immunization with a vaccine is considered an effective measure to control these diseases. However, it is still unknown whether PCV2 vaccines have protective immune responses on the animals infected with swine influenza virus (SIV), a pandemic virus in swine herds. In this study, we first compared the effects of 2 different PCV2 vaccines on normal mice and SIV-infected mice, respectively. The results showed that these two vaccines had protective immune responses in normal mice, and the subunit vaccine (vaccine S) had better effects. However, the inactivated vaccine (vaccine I) instead of vaccine S exhibited more immune responses in the SIV-infected mice. SIV infection significantly decreased the immune responses of vaccine S in varying aspects including decreased PCV2 antibody levels and increased PCV2 replication. Mechanistically, further studies showed that SIV infection increased IL-10 expression and M2 macrophage percentage, but decreased TNF-α expression and M1 macrophage percentage in the mice immunized with vaccine S; on the contrary, macrophage depleting by using clodronate-containing liposomes significantly alleviated the SIV infection-induced decrease in the protective immune responses of vaccine S against PCV2. This study indicates that SIV infection decreases the protective immune responses of vaccine S against PCV2. The macrophage polarization induced by SIV infection might facilitate decreased immune responses to vaccine S, which provides new insight into vaccine evaluation and a reference for the analysis of immunization failure.

## Introduction

Porcine circovirus type 2 (PCV2) belongs to the *Circoviridae* family and is a single-standard circular DNA virus. As a subtype with pathogenicity, PCV2 has been proved to be the primary etiological agent that cause porcine circovirus associated diseases and post weaning multisystemic wasting syndrome (Hamel et al., 1998; Opriessnig and Meng, 2007), resulting in serious economic losses (Chae, 2005; Baekbo et al., 2012). In addition, PCV2 has also been proven to be immunosuppressive, which increases the risk of the host infecting other pathogens (Segalés, 2012). Therefore, it is valuable to utilize effective measures to control the PCV2 infection in pigs.

Up to now, vaccination has been always considered one of the most efficient methods of preventing PCV2 infection in pigs. However, the majority of PCV2 vaccine trials were conducted in healthy animals (Wang et al., 2013; Zhang et al., 2017), which is inconsistent with field conditions. The potential infection of other pathogens has always existed in swine herds and is likely to weaken immune responses to PCV2 vaccines. Swine influenza virus (SIV), an especially common virus, is a primary pathogen causing swine flu, an acute respiratory infection with clinical symptoms including coughing, sneezing, nasal discharge, fever, lethargy, and decreased appetite. Its morbidity in pigs can reach 100%, and mortality is generally low (Thacker and Janke, 2008; Pearce et al., 2012). However, its impact on pigs is not negligible because the latent and universality of this virus makes it difficult to be discovered in a timely fashion, thereby greatly influencing the programmed vaccination in pigs. Moreover, SIV is also an immunosuppressive virus. A previous study reported that it could promote alveolar macrophage polarization into immunosuppressive phenotype (Zhao et al., 2014). Therefore, SIV infection may increase PCV2 infection via inducing immunosuppression, but this has not been reported to date.

As the first line of defense, alveolar macrophages have been proven to play an indispensable role in resisting the invasion of SIV (Christoph et al., 2014; Cardani et al., 2017). Alveolar macrophages, a kind of macrophages existing in the lungs, play key roles in innate immunity (Wynn et al., 2013; Byrne et al., 2016). In general, they are classified as two phenotypes: M1 macrophages, which promote inflammatory responses and tissue injury; and M2 macrophages, which inhibit inflammation and promote tissue repair (Byrne et al., 2016; Wynn and Vannella, 2016). Macrophage polarization refers to an estimate of macrophage activation at a given point in space and time, which is a dynamic process and usually associated with inflammatory conditions (Murray, 2017). Multiple growth factors and cytokines including TNF-α and IL-10 can be markers for distinguishing the M1/M2 polarization state (Patel et al., 2016). Toll-like receptors (TLRs) as important components of the pattern recognition receptors, have been found in 13 members (TLR1-TLR13) in mammals, and play an indispensable role in innate immune responses (Fabris et al., 2017). TLR4 is a receptor on the cell membrane that presents a variety of cells, including alveolar macrophages. LPS or viruses can be recognized by TLR4 and then activate innate immune responses and promote inflammation, which is a protection mechanism defense against pathogens (Lu et al., 2008). However, the overexpression of TLR4 often leads to chronic inflammatory disorders and tissue damage *in vivo* (Loretta et al., 2012; Xu et al., 2017), which may weaken immune responses to vaccination. Therefore, in the present study, we examined whether SIV infection could induce excessive inflammation to result in immunosuppression, thereby attenuating the protective immune responses of PCV2 vaccines, providing new insights into vaccine evaluation and a reference for the analysis of immunization failure.

## Materials and Methods

### Ethics Statement

The research protocol for this study was approved by the Ethics Committee for Animal Experimentation of Nanjing Agricultural University (approval number: SYXK-SU-2011-0036). All animal care and use procedures were conducted in strict accordance with the Animal Research Committee guidelines of the College of Veterinary Medicine at Nanjing Agricultural University, and all efforts were made to minimize animal suffering and to reduce the number of animals used.

### Viruses and Vaccines

Swine influenza virus (SIV) strain A/swine/Guangxi/18/2011 (H1N1) was kindly provided by Harbin Veterinary Research Institute, Chinese Academy of Agricultural Sciences (Harbin, China). The virus was cultivated in MDCK cells, with serum free medium supplemented with 1 μg/mL TPCK-treated trypsin, in 5% CO_2_, 37°C condition. The 50% tissue culture infectious doses (TCID_50_) were determined by observing the cytopathic effects at 48 h post-infection. Porcine circovirus type 2 (PCV2NJ2002, PCV2b) was stored in our laboratory (Liu et al., 2017). The virus was cultivated in PK-15 cells, with the Dulbecco’s modified eagle medium (DMEM) containing 2% newborn bovine serum free-medium, in 5% CO_2_, 37°C condition (Zhai et al., 2019). TCID_50_ was determined by indirect immunofluorescence.

PCV2 vaccine I is a commercial inactivated vaccine (SH strain, No. zycp-ym003, O/W adjuvant) purchased from Jiangsu Nannong Hi-Tech Co., Ltd. (Nanjing, China), and the vaccine S was a commercial subunit vaccine (ZJ/c strain, Ingelvac CircoFLEX^®^, O/W adjuvant) imported from Boehringer-Ingelheim Animal Health Co., Ltd.

### Animal Experiments

The female BALB/c mice (6–8 weeks old) were obtained from the Yangzhou University Experimental Animal Center. All of the animals were monitored at a controlled temperature under a 12 h light/dark cycle with enough standard rodent chow and water for 1-week adaptation. BALB/c mice were challenged with H1N1 SIV as the SIV-infected model, which has been reported as an effective tool for viral replication and pathogenesis *in vivo* (Xin et al., 2015; Qian et al., 2017; Park et al., 2018), and 2 different PCV2 vaccines were immunized on normal mice and the SIV-infected mice for comparison, respectively.

Experiment 1: 80 mice were divided into 8 groups randomly as followed: (a) Blank group; (b) PCV2 control group; (c) SIV control group; (d) SIV and PCV2 control group; (e) Vaccine I and PCV2 group; (f) Vaccine S and PCV2 group; (g) SIV, vaccine I and PCV2 group; (h) SIV, vaccine S and PCV2 group. Mice from c, d, g and h groups were challenged via nasal infection with 1000 TCID_50_ (TCID_50_ = 10^–4⋅5^) SIV at 1st day, and the negative controls were challenged with equivalent doses of PBS. PCV2 vaccines were immunized via intraperitoneal injection with 0.2 mL for each mouse on the fourth day, and the immunization period lasted 21 days. Then, PCV2 was given via intraperitoneal injection with 1000 TCID_50_ (TCID_50_ = 10^–6^) for each mouse in b, d, e, f, g, and h groups, and the negative control mice were injected with equivalent doses of PBS. The mice challenged with viruses were kept in isolation. At 14 d post-PCV2 infection, all of the animals were euthanized, bronchoalveolar lavage (BAL) fluid, whole blood, lung, thymus, and spleen tissues were collected.

Experiment 2: 20 mice were randomly divided into two groups as follows: (a) SIV + vaccine S + PCV2 + clodronate liposomes group (Clo-lip group); (b) SIV + vaccine S + PCV2 + PBS liposomes group (PBS-lip group). Alveolar macrophages were specifically depleted by intranasally injecting clodronate-containing liposomes according to the instructions of the Clodronate Liposome kit (LIPOSPMA, Shanghai, China). In brief, the mice in the Clo-lip group were challenged via intranasal infection with 50 μL clodronate-containing liposomes every 4 days, and the control mice (PBS-lip group) were administrated with equivalent doses of PBS liposomes at the same time. The administration procedures for SIV, PCV2, and PCV2 vaccines were the same with “Experiment 1.” At the end of this experiment, BAL fluid, thymus, and spleen tissues were collected.

### Measurements of Antibody Levels

The serum was collected at 14 and 21 days post-immunization, respectively. The levels of anti-PCV2 specific antibodies were measured by using a sandwich enzyme-linked immunosorbent assay. In brief, the purified recombinant cap protein was coated on 96-well plates for the enzyme-labeled reaction. After antibody incubation and TMB coloration, the OD value was obtained at a wavelength of 450 nm, and the P/N > 2.1 was considered to be positive.

### Quantitative Real-Time PCR

The DNA was extracted from spleens, using the TaKaRa DNA Mini kit (TaKaRa, Dalian, China). The purified DNA was used as a template for qPCR amplification, and a 117-bp fragment from the PCV2 ORF2 gene was amplified with specific primers (forward primer 5′-TAGTATTCAAAGGGCACAG-3′, reverse primer 5′-AAGGCTACCACAGTCAG-3′). A recombinant pMD19 plasmid vector (TaKaRa) containing a PCV2 genome insert was used as a positive control.

Total RNA was extracted from lungs to determine the relative mRNA level of SIV M protein, and from spleens and lungs to detect the relative TLR4, TNF-α mRNA and IL-10 mRNA levels. Target and reference gene primers were designed and synthesized according to the known sequences. Quantitative real-time PCR was conducted using a TaKaRa SYBR Green real-time PCR kit (SYBR Premix Ex Taq) and the ABI Prism Step One Plus detection system (Applied Biosystems, Foster City, United States). Finally, the relative mRNA levels of target genes were calculated by the 2^–ΔΔCT^ method with GAPDH as a reference gene.

### Western Blotting Analysis

Lungs were collected to detect the relative expression of SIV NP protein. In brief, total proteins were extracted, and then the protein concentration was measured using a BCA kit (Beyotime, China). Subsequently, the proteins were denatured, subjected to SDS-PAGE, and then transferred to the PVDF membranes. Next, the membranes were blocked with 5% BSA, followed by overnight incubation at 4°C in anti-NP antibody (Abcam, United Kingdom) and 2 h of incubation with an anti-mouse secondary antibody (Cell Signaling Technology, United States) at RT. Finally, the bound antibodies were visualized using an enhanced chemiluminescence kit (Beyotime, China).

### Histopathological Analysis

Spleens and lungs were collected from mice and fixed in 4% neutral-buffered formalin solution. The samples were then embedded in paraffin to be further cut in 4 μm sections for hematoxylin and eosin (HE) staining. The tissue sections were observed and photographed under an optical microscope. The degree of spleen damage was assessed based on the amount of white pulp atrophy, structure disorder, and hemorrhage, and the degree of lung damage was evaluated according to the amount of edema, septal thickening, and cellular infiltration.

### Immunohistochemical Analysis

The fresh spleens were collected and fixed in 4% neutral buffered formalin for IHC staining. In brief, tissue sections were incubated with the specific primary antibodies (anti-cap or anti-TLR4 antibodies) at 37°C for 1 h. After washing in PBS, the sections were incubated with a horseradish peroxidase labeled anti-mouse antibody at 37°C for 1 h. Next, freshly prepared DAB was added into the sections at RT for 5 min. Finally, after hematoxylin staining for 1 min, the sections were dehydrated and mounted by neutral gum, and then examined by an optical microscope. Images were captured with a Pannoramic viewer (Pannoramic MIDI, 3D HISTECH), and data were analyzed using DensitoQuant software (QuantCenter, 3DHISTECH). A histochemistry score (H-score) was calculated as previously described (Sun et al., 2019).

### Bronchoalveolar Lavage Fluid Collection and Macrophage Classification

BAL was conducted to obtain alveolar macrophages from fresh mouse lungs. In brief, each lung was repeatedly flushed with PBS, and cells were harvested from the BAL fluid by centrifugation. Cells from the BAL fluid were incubated with an Fc receptor blocker (BD Biosciences) to reduce non-specific binding and then incubated with specific F4/80-APC, CD11b-PerCP-Cy5.5, CD80-PE, and CD206-FITC antibodies (eBioscience; BD Biosciences) for 30 min at 4°C (Sun et al., 2018). Subsequently, flow cytometry (BD Biosciences) was used to collect and classify the alveolar macrophages, and then data were analyzed using FlowJo for Mac 10.7 (Tree Star, Inc.).

### Statistical Analysis

All statistical analyses were conducted using GraphPad Prism 6. Student’s two-tailed *t*-test in two groups and one-way or two-way ANOVA in multiple groups were used, and the results were expressed as the mean ± standard error (S.E.). *P* < 0.05 was considered statistically significant.

## Results

### The Protective Immune Responses of 2 Porcine Circovirus Type 2 Vaccines on Normal Mice

**FIGURE 1:**
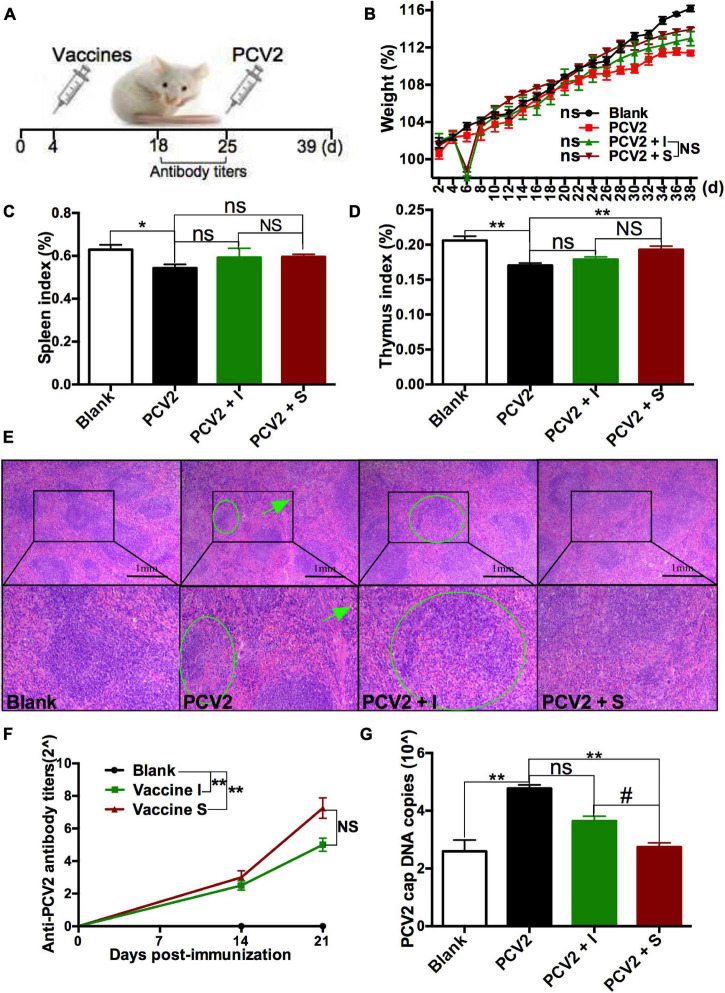
Effects of 2 different PCV2 vaccines on normal mice. **(A)** Mice were immunized with subunit vaccine (vaccine S) and inactivated vaccine (vaccine I) at day 4, and then were injected with PCV2 at day 25. **(B)** Weight gain expressed as the percentage of initial weight. **(C)** Spleen index and **(D)** thymus index were calculated by the ratio of spleen or thymus to body weight. **(E)** HE staining for spleens. The green circle and arrow indicated white pulp structure disorder and hemorrhage, respectively. **(F)** PCV2-specific antibody levels in serum were assessed at days 14 and 21 post-immunization. **(G)** The PCV2 DNA copies in spleen. Data were presented as means ± SEM of mice (*n* ≥ 3) in each group. Compared with PCV2 group, *P < 0.05, **P < 0.01, and ns, not significant; Compared with PCV2 + vaccine I group, ^#^*P* < 0.05 and NS, not significant.

To explore the immune responses of different PCV2 vaccines on normal animals, mice were treated with subunit vaccine (vaccine S) and inactivated vaccine (vaccine I), respectively, and then challenged by PCV2 at 21 days post-immunization (Figure 1A). As expected, no obvious clinical symptoms and abnormal phenomena were observed among all mice. Weight was measured every 2 days, and the results showed that no significance was observed among all mice (Figure 1B). Subsequently, spleen index (spleen/body weight percentage) and thymus index (thymus/body weight percentage) were calculated to evaluate the immune organ damage. As demonstrated in Figures 1C,D, PCV2 infection significantly decreased the spleen and thymus index, while vaccine S markedly alleviated the decrease in thymus index induced by PCV2 infection, and no significance was observed between the PCV2 group and PCV2 + vaccine I groups. Similar results were demonstrated by HE staining for spleens. As shown in Figure 1E, white pulp atrophy, structure disorder, and hemorrhage were observed in the spleens of mice from the PCV2 group, while these pathological damages were relieved by vaccine S.

The PCV2-specific antibody levels in serum were detected at 14 and 21 days post-immunization, respectively, to assess the immune responses of 2 PCV2 vaccines. As shown in Figure 1F, higher levels of the PCV2-specific antibodies were produced in vaccine I and vaccine S groups than that in the blank group at 21 days post-immunization, while no significance was observed between vaccine I and S groups. To more accurately assess the immune responses of the 2 vaccines, PCV2 replication was also measured. As indicators to assess PCV2 replication, the PCV2 DNA copies were detected using qRT-PCR, and cap protein was stained for IHC examination. The results showed that PCV2 infection significantly increased PCV2 DNA copies, but the increase was markedly down-regulated by vaccine S, not vaccine I, and PCV2 DNA copies were lower in the PCV2 + vaccine S group than those in the PCV2 + vaccine I group (Figure 1G). Similarly, the cap-positive staining (H-score) significantly reduced in 2 vaccine groups compared with the PCV2 control group, and the inhibitory effect was more obvious in PCV2 + vaccine S than that in the PCV2 + vaccine I group (Supplementary Figure 1).

Taken together, the vaccine S induced higher levels of PCV2-specific antibodies, significantly reduced PCV2 replication, mitigated the organ damage caused by PCV2 infection, suggesting that it produced protective immune responses on normal PCV2-infected mice. In terms of reducing PCV2 DNA copies, it also had better immune responses compared to vaccine I.

### The Protective Immune Responses of 2 Porcine Circovirus Type 2 Vaccines on the Swine Influenza Virus-Infected Mice

**FIGURE 2:**
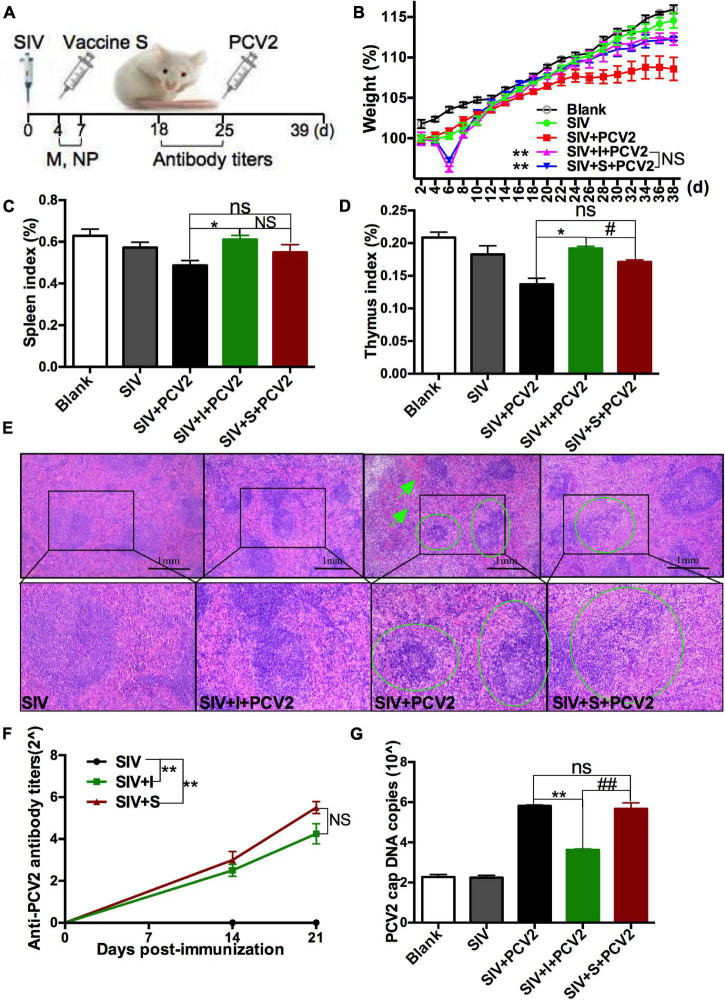
Effects of 2 different PCV2 vaccines on the SIV-infected mice. **(A)** Mice were intranasally infected with SIV at day 0, immunized with 2 PCV2 vaccines at day 4, and then injected with PCV2 at day 25. **(B)** Weight gain expressed as the percentage of initial weight. **(C)** Spleen index and **(D)** thymus index were calculated by the ratio of spleen or thymus to body weight. **(E)** HE staining for spleens. The green circle and arrow indicated white pulp structure disorder and hemorrhage, respectively. **(F)** PCV2-specific antibody levels in serum were assessed at days 14 and 21 post-immunization. **(G)** The PCV2 DNA copies in spleen. Data were presented as means ± SEM of mice (*n* ≥ 3) in each group. Compared with SIV + PCV2 group, *P < 0.05, **P < 0.01, and ns, not significant; Compared with SIV + PCV2 + vaccine I group, ^#^*P* < 0.05, ^##^*P* < 0.01, and NS, not significant.

To explore the role SIV infection played in the protective immune responses of PCV2 vaccines, SIV was injected to mice as described in Experiment 1 (Figure 2A), and then the immune responses of 2 PCV2 vaccines on the SIV-infected mice were compared. At first, relative SIV M mRNA (Supplementary Figure 2A) and NP expression (Supplementary Figure 2B) levels, as well as lung damage (Supplementary Figure 2C) in the SIV control group were measured to confirm SIV infection. The results showed that the SIV infection model had been successfully established. Next, body weight gain, spleen index, and thymus index were measured. The results demonstrated that the body weight gain significantly increased in 2 vaccine groups relative to the SIV + PCV2 control group (Figure 2B). As shown in Figures 2C,D, there was significance in both thymus index and spleen index between vaccine I, not vaccine S and SIV + PCV2 control groups; moreover, thymus index was higher in vaccine I group than that in vaccine S group. After SIV and PCV2 infection, white pulp atrophy, structure disorder, and hemorrhage were observed in the spleens of mice, while these pathological damages were relieved by 2 vaccines, and vaccine I had a better effect (Figure 2E).

The PCV2-specific antibody titers were detected after SIV infection and vaccine immunization. The results showed that the antibody titers markedly increased in SIV + vaccine S and SIV + vaccine I groups relative to the SIV control group, but there was no significance between SIV + vaccine S and SIV + vaccine I groups (Figure 2F). As above mentioned, PCV2 replication was also evaluated. As shown in Figure 2G, vaccine I instead of vaccine S significantly decreased the PCV2 DNA copies in the SIV + PCV2 control group, and the PCV2 DNA copies in SIV + PCV2 + vaccine I group were lower than those in SIV + PCV2 + vaccine S. The same tendency was observed in the cap-positive staining (H-score) (Supplementary Figure 3).

In summary, these data suggest that vaccine I instead of vaccine S produced adequate immune responses on the SIV-infected mice.

### Swine Influenza Virus Infection Decreased the Protective Responses of Vaccine S, Increased Toll-Like Receptors 4 Expression, and Promoted Alveolar Macrophage Polarization From M1 to M2

**FIGURE 3:**
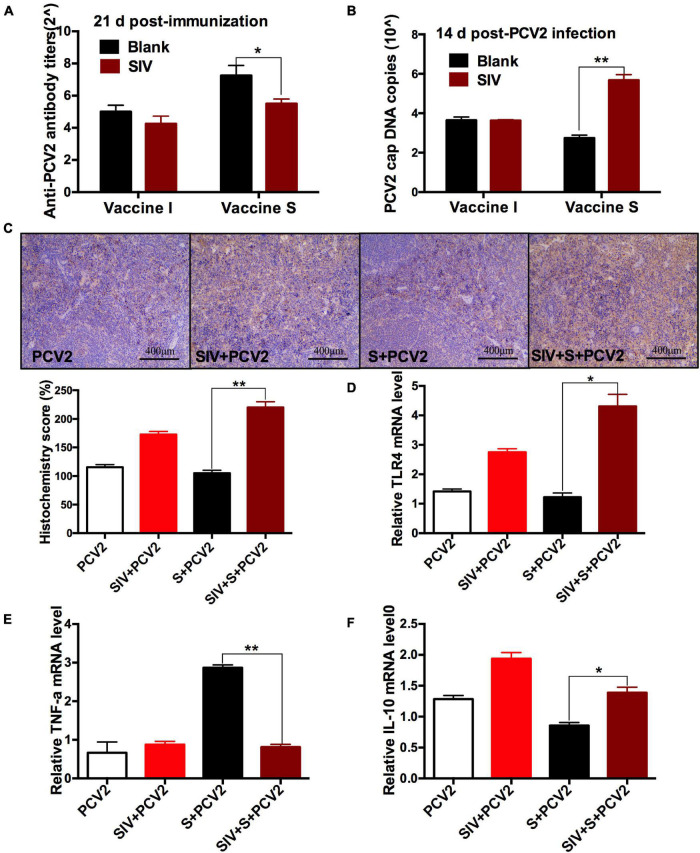
The effects of SIV infection on the immune responses of vaccine S. **(A)** PCV2-specific antibody levels in serum were assessed at day 21 post-immunization. **(B)** The PCV2 DNA copies in spleen. **(C)** Immunohistochemical (IHC) analysis for the TLR4 expression in spleens. The TLR4-specific staining intensity was showed by the histochemistry score (H-score). **(D)** The relative TLR4 mRNA level in spleens. **(E)** The relative TNF-α mRNA level in lungs. **(F)** The relative IL-10 mRNA level in lungs. Data were presented as means ± SEM of mice in each group. Data were presented as means ± SEM of mice (*n* ≥ 3) in each group. *P < 0.05, **P < 0.01.

To further evaluate the effect of SIV infection on the immune responses of 2 vaccines, the PCV2-specific antibody titers and cap DNA copies were compared between before and after SIV infection. As shown in Figure 3A, after SIV infection, the antibody levels of the vaccine S group other than the vaccine I group at 21 days post-immunization were significantly reduced. Meanwhile, the PCV2 DNA copies in the SIV-infected mice immunized by vaccine S instead of vaccine I were significantly increased (Figure 3B). These results together suggest that SIV infection significantly weakened the immune responses of vaccine S.

**FIGURE 4:**
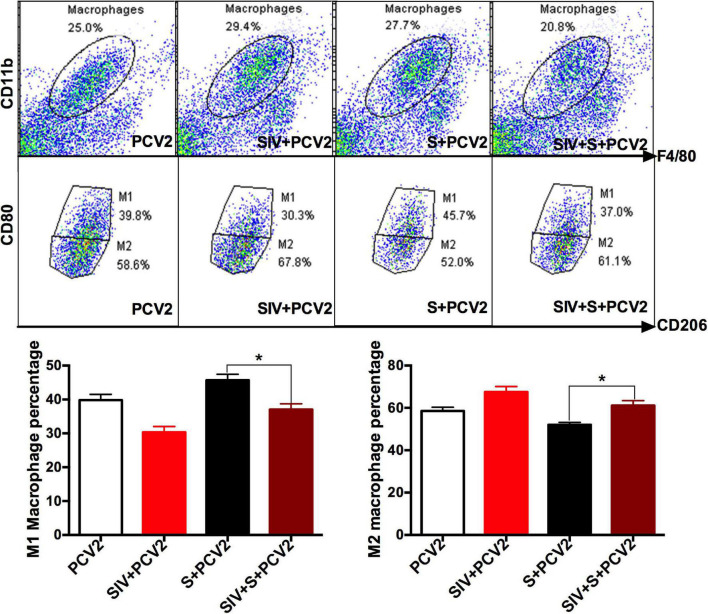
SIV promoted macrophage polarization from M1 to M2. Bronchoalveolar lavage (BAL) fluid was detected using a flow cytometer. CD11b and F4/80 were used for marking macrophages; CD80 and CD206 were markers for M1 and M2 macrophages, respectively. The proportion of M1 and M2 macrophages in the total population of macrophages. Data were presented as means ± SEM of mice (*n* ≥ 3) in each group. *P < 0.05.

To explore the reasons why vaccine S was weakened by SIV infection, TLR4, inflammation-related cytokines, and macrophage phenotype were further detected. As expected, TLR4-positive staining (Figure 3C) and its mRNA level (Figure 3D) significantly enhanced in the PCV2 + vaccine S + SIV group relative to the PCV2 + vaccine S group. Moreover, SIV infection significantly decreased TNF-α mRNA level (Figure 3E) but enhanced the IL-10 mRNA level in the PCV2 + vaccine S group (Figure 3F). As mentioned in the “Introduction” section, TNF-α and IL-10 are the markers for M1 and M2 macrophages, respectively. Therefore, our results indicated that SIV infection might promote macrophage polarization from M1 to M2. To further verify this conclusion, BAL fluid was collected to detect the alveolar macrophage phenotype considering that the lung was the target organ to infect SIV. The results showed that the mice in the PCV2 + vaccine S group showed a markedly decreased M1 macrophage percentage but significantly increased M2 macrophage percentage after SIV infection (Figure 4).

Taken together, these results suggest that SIV infection decreased the protective effects of vaccine S and promoted TLR4 expression and alveolar macrophage polarization from M1 to M2, which could cause the immunization failure of vaccine S on the SIV-infected mice.

### Macrophage Depletion Alleviated the Swine Influenza Virus Infection-Induced Decrease in the Protective Immune Response of Vaccine S

**FIGURE 5:**
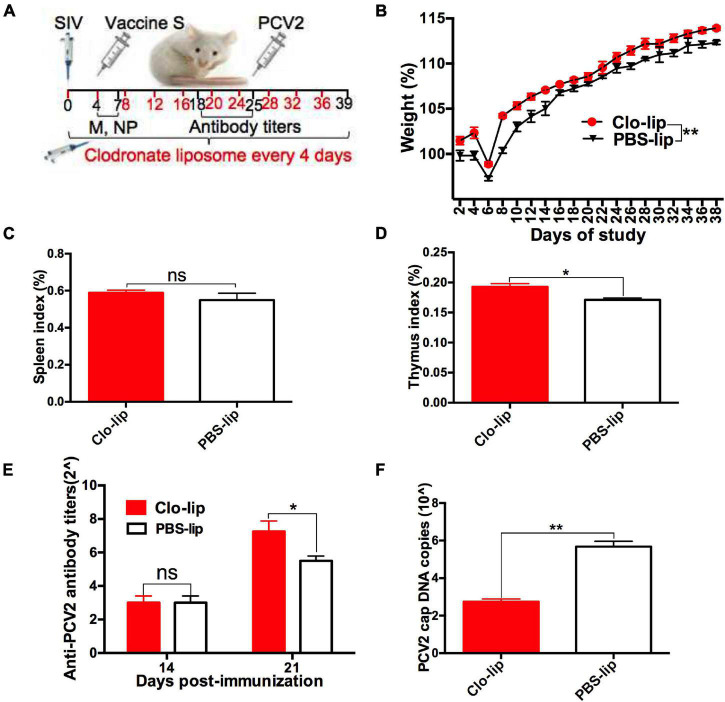
Alveolar macrophage depletion attenuated the SIV-induced decrease in the protective immune effect of vaccine S. **(A)** Mice were intranasally infected with SIV at day 0, injected with clodronate-containing liposomes (Clo-lip) or PBS liposomes (PBS-lip) every 4 days, immunized with vaccine S at day 4, and then injected with PCV2 at day 25. **(B)** Weight gain expressed as the percentage of initial weight. **(C)** Spleen index and **(D)** thymus index were calculated by the ratio of spleen or thymus to body weight. **(E)** PCV2-specific antibody levels in serum were assessed at days 14 and 21 post-immunization. **(F)** The PCV2 DNA copies in spleen. Data were presented as means ± SEM of mice (*n* ≥ 3) in each group. *P < 0.05, **P < 0.01, and ns, not significant.

To further investigate whether SIV infection decreased the protective responses of vaccine S via promoting alveolar macrophage polarization from M1 to M2, clodronate liposome, a macrophage scavenger was used to specifically deplete lung macrophages as described in “Experiment 2” (Figure 5A). In order to evaluate the depletion efficiency of Clo-lip, a preliminary test was conducted for a period of 39 days, and the total number of alveolar macrophages in each BAL sample from the Clo-lip group and PBS-lip group was calculated every day as previously described (Garcia-Castillo et al., 2020).

As expected, the number of BAL macrophages decreased by about 80% after one CLP injection, and then was almost exhausted after multiple CLP injections (Supplementary Figure 4A). These results suggested that CLP injection was useful for depleting alveolar macrophages. Next, weight gain (Figure 5B), spleen index (Figure 5C), thymus index (Figure 5D), anti-PCV2 antibody titers (Figure 5E), PCV2 DNA copies (Figure 5F), and cap-positive staining (Supplementary Figure 4B) were measured and compared between Clo-lip and PBS-lip groups. The results showed that weight gain, thymus index, and anti-PCV2 antibody titers significantly increased after macrophage depletion, while PCV2 replication markedly decreased as demonstrated by decreased PCV2 DNA copies and H-score. Taken together, these results demonstrated that macrophage depletion alleviated the SIV infection-induced decrease in the protective immune responses of vaccine S, which suggested that SIV infection might decrease the protective immune responses of vaccine S via promoting alveolar macrophage polarization from M1 to M2.

## Discussion

As an effective measure to prevent pigs from PCV2-related diseases (Opriessnig et al., 2010; Seo et al., 2012), it was important to ensure the effective immune responses of PCV2 vaccines under field conditions. Evidence indicates that most of the available vaccines failed to effectively decrease the mortality of PCV2 infection in herds due to a variety of factors (Beach and Meng, 2012). Of which, the widespread SIV in pigs was considered as a potential reason for immunization failure (Choi et al., 2002). However, the previous PCV2 vaccine evaluation tests were always conducted on healthy animals including piglets (Seo et al., 2014; Huan et al., 2018; Zhang et al., 2018), which made it uncertain whether PCV2 vaccines could effectively work under field conditions. In this present study, the immune responses of two kinds of PCV2 vaccines on SIV-infected mice were tested to imitate a commercial farming condition.

Given that the immune responses of PCV2 vaccines could not be reflected because of invisible clinical symptoms (Deng et al., 2013; Wang et al., 2017), their effects were evaluated based on the growth performance of mice, anti-PCV2 antibody levels, and PCV2 replication *in vivo*. Our results showed that 2 PCV2 vaccines had protective immune responses on normal mice, but the effects of vaccine S were better than those of vaccine I, as demonstrated by the significantly decreased PCV2 DNA copies and organ damage. These results suggested that vaccine S could have more application value than vaccine I on normal mice. In contrast, vaccine I instead of vaccine S produced adequate immune responses on the SIV-infected mice. SIV infection weakened the immune responses of vaccine S, as demonstrated by the significantly decreased anti-PCV2 antibody levels and increased PCV2 replication, which meant that vaccine S only played adequate protective roles in animals without pathogen infection, and there might be a risk of immunization failure due to latent SIV infection. In summary, our results confirmed that the immune responses of vaccine S were inconsistent between the normal and SIV-infected mice.

We try to explain the reason why vaccine S was more easily influenced by SIV than vaccine I. In general, immune responses to vaccination include neutralizing antibodies and cell-mediated immunity (Afghah et al., 2017). The inactivated vaccine often fails to produce sufficient cell-mediated immunity due to the inactivated virus. But the subunit vaccine can increase CD4^+^ and CD8^+^ levels thereby producing long-lasting protection (Ferrari et al., 2014), which plays an important role in the immune responses of the subunit vaccine. This difference in action mechanism between inactivated vaccine and subunit vaccine may be one of the reasons for the instability of vaccine S. Moreover, a previous study has reported that the porcine reproductive and respiratory syndrome virus had an adverse effect on naive T cells thereby influencing the immune responses of PCV2 vaccines (Canelli et al., 2016). Given the importance of cell-mediated immunity in the immune responses of subunit vaccines, we concluded that the immunization failure of vaccine S may be related to the immune organ damage or immunosuppression induced by SIV infection.

As our results showed, increased TLR4 expression was detected in the spleens of the mice infected with SIV, and the elevation of TLR4 for at least 5 weeks after SIV infection, which meant that the innate immune responses were continuously activated to resist viral infection. More and more studies have confirmed that the overexpression or sustained activation of TLR4 could lead to excessive inflammation or tissue damage *in vivo* (Perrone et al., 2008; Tate et al., 2010). Consistently, the SIV-infected mice showed more pronounced thymus damage than the control mice, which further indicates that the adverse effects of SIV infection might be associated with the increased expression of TLR4.

SIV usually invades the body through the respiratory tract and directly acts on alveolar macrophages. Subsequently, macrophages manifest as M1 phenotype to defense against the pathogen at the initial stage of infection and then change into M2 macrophages (Patel et al., 2016). Our data suggested that SIV infection induced the macrophage polarization from M1 to M2, meaning the macrophages exhibited a more stable suppression following excessive inflammatory responses. Due to the compensatory effects and complexity of the body, the immune system of the whole body may be affected, resulting in insufficient responses to antigens and failing to produce effective immune protection. Therefore, the immunization failure of vaccine S in the presence of SIV may be related to the immunosuppression as demonstrated by the alveolar macrophage polarization from M1 to M2 following excessive inflammatory responses. However, studies on the relationship between TLR4 activation and alveolar macrophage polarization from M1 to M2, in this case, remain to be further performed.

## Conclusion

In conclusion, PCV2 vaccine S is more effective in defense against PCV2 infection on normal mice, but PCV2 vaccine I instead of vaccine S is better on the SIV-infected mice. SIV infection significantly weakens the protective immune responses of vaccine S, and the risk of immunization failure of the PCV2 vaccine might increase due to the pandemic SIV in herds. These results provided a feasible reference for the clinical analysis of the immunization failure of different vaccines.

## Data Availability Statement

The original contributions presented in the study are included in the article/Supplementary Material, further inquiries can be directed to the corresponding author/s.

## Ethics Statement

The animal study was reviewed and approved by the Ethics Committee for Animal Experimentation of Nanjing Agricultural University.

## Author Contributions

YS, YZ, and KH designed this project, wrote, and revised the manuscript. YS, JZ, and ZL conducted the experiments. All authors reviewed the manuscript.

## Conflict of Interest

The authors declare that the research was conducted in the absence of any commercial or financial relationships that could be construed as a potential conflict of interest.

## Publisher’s Note

All claims expressed in this article are solely those of the authors and do not necessarily represent those of their affiliated organizations, or those of the publisher, the editors and the reviewers. Any product that may be evaluated in this article, or claim that may be made by its manufacturer, is not guaranteed or endorsed by the publisher.

## Supplementary Material

Supplementary Figure 1 IHC analysis for the cap expression in spleens of normal mice. The cap-specific staining intensity was showed by H-score. Compared with PCV2 group, ***P* < 0.01; Compared with vaccine I group, NS, not significant.

Supplementary Figure 2 SIV infection measurement. **(A)** SIV M mRNA level, **(B)** NP protein level and **(C)** lung damage were measured to verify SIV infection. The yellow arrow indicated hemorrhage, and the green arrow indicated inflammatory cell infiltration. Data were presented as means ± SEM of mice (*n* ≥ 3) in each group. **P* < 0.05, ***P* < 0.01, and ns, not significant.

Supplementary Figure 3 IHC analysis for the cap expression in spleens of SIV-infected mice. The cap-specific staining intensity was showed by H-score. Compared with PCV2 group, ***P* < 0.01 and ns, not significant; Compared with vaccine I group, ^##^*P* < 0.01.

Supplementary Figure 4 Macrophage numbers and cap expression. **(A)** The number of macrophages from BAL sample was detected and calculated according to the presented time point. **(B)** IHC analysis for the cap expression in spleens. The cap-specific staining intensity was showed by the H-score. Data were presented as means ± SEM of mice (*n* ≥ 3) in each group. ***P* < 0.01.
