# Supplementary figures and images for: Swine Influenza Virus Infection Decreases the Protective Immune Responses of Subunit Vaccine Against Porcine Circovirus Type 2
Source: Front Microbiol. 2021 Dec 24;12:807458. doi: 10.3389/fmicb.2021.807458 (PMC8740023; doi:10.3389/fmicb.2021.807458)

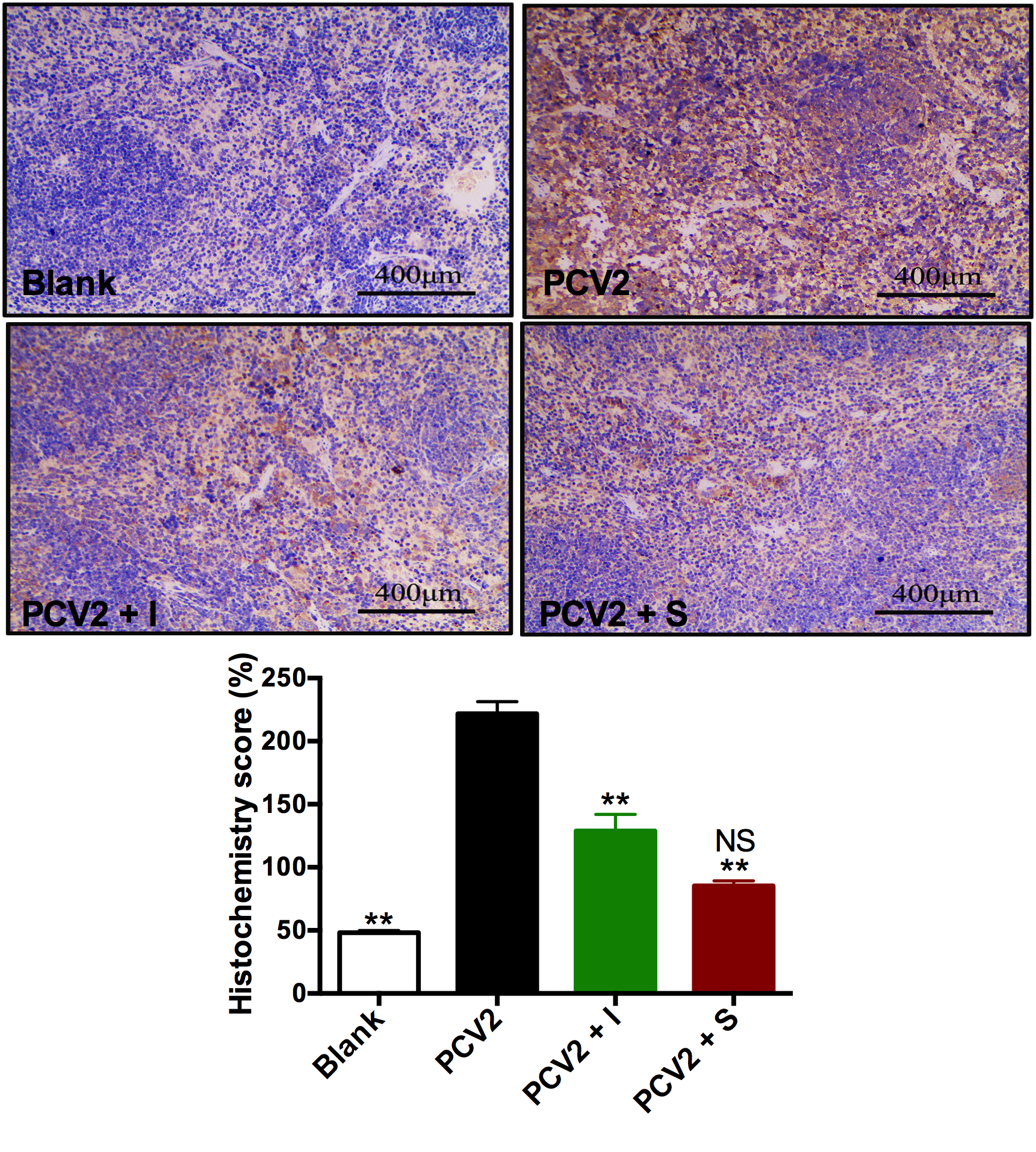

Supplement: Supplementary Figure 1 — IHC analysis for the cap expression in spleens of normal mice. The cap-specific staining intensity was showed by H-score. Compared with PCV2 group, **P < 0.01; Compared with vaccine I group, NS, not significant. [file Image_1.TIFF]

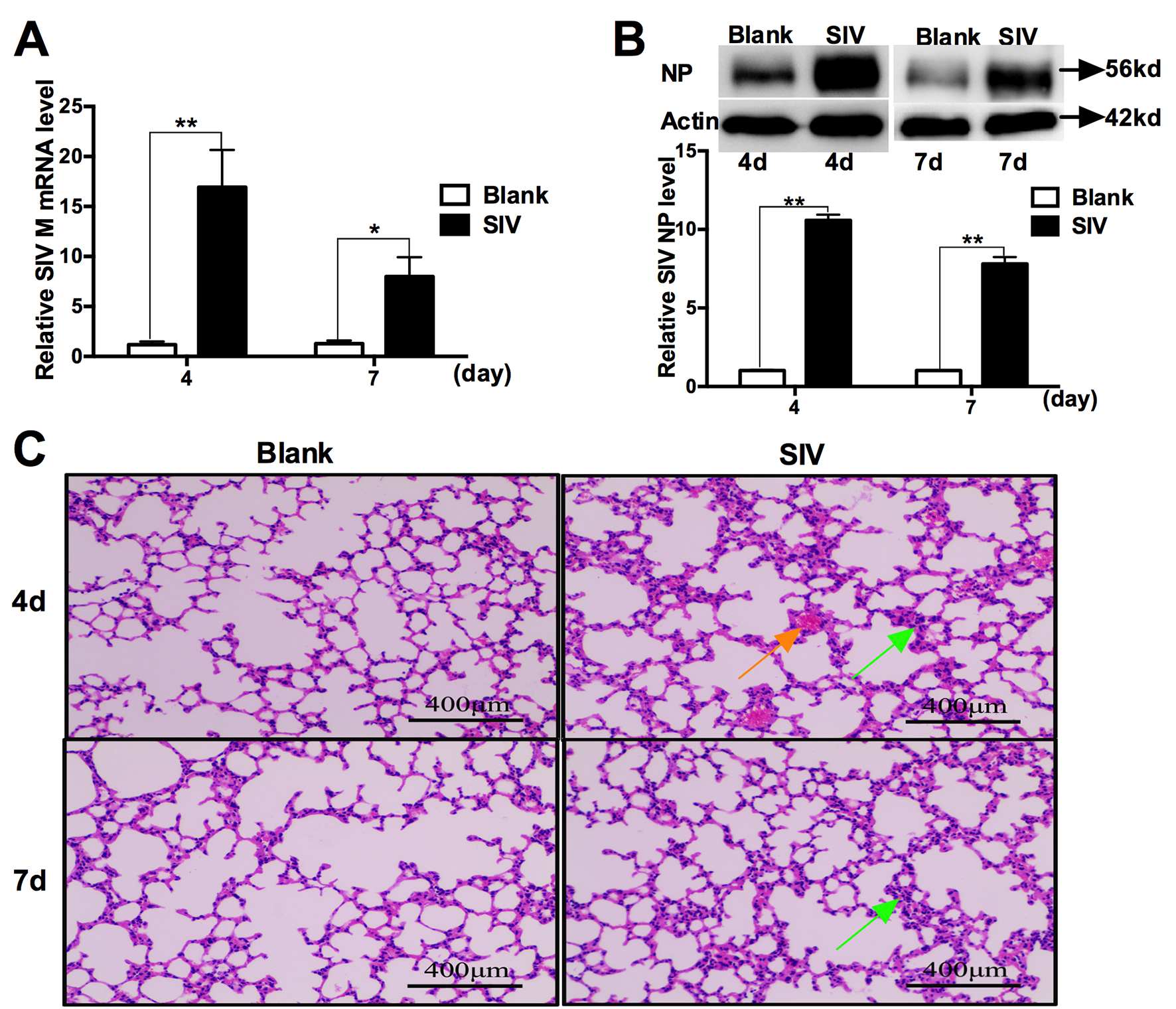

Supplement: Supplementary Figure 2 — SIV infection measurement. (A) SIV M mRNA level, (B) NP protein level and (C) lung damage were measured to verify SIV infection. The yellow arrow indicated hemorrhage, and the green arrow indicated inflammatory cell infiltration. Data were presented as means ± SEM of mice (n ≥ 3) in each group. *P < 0.05, **P < 0.01, and ns, not significant. [file Image_2.TIFF]

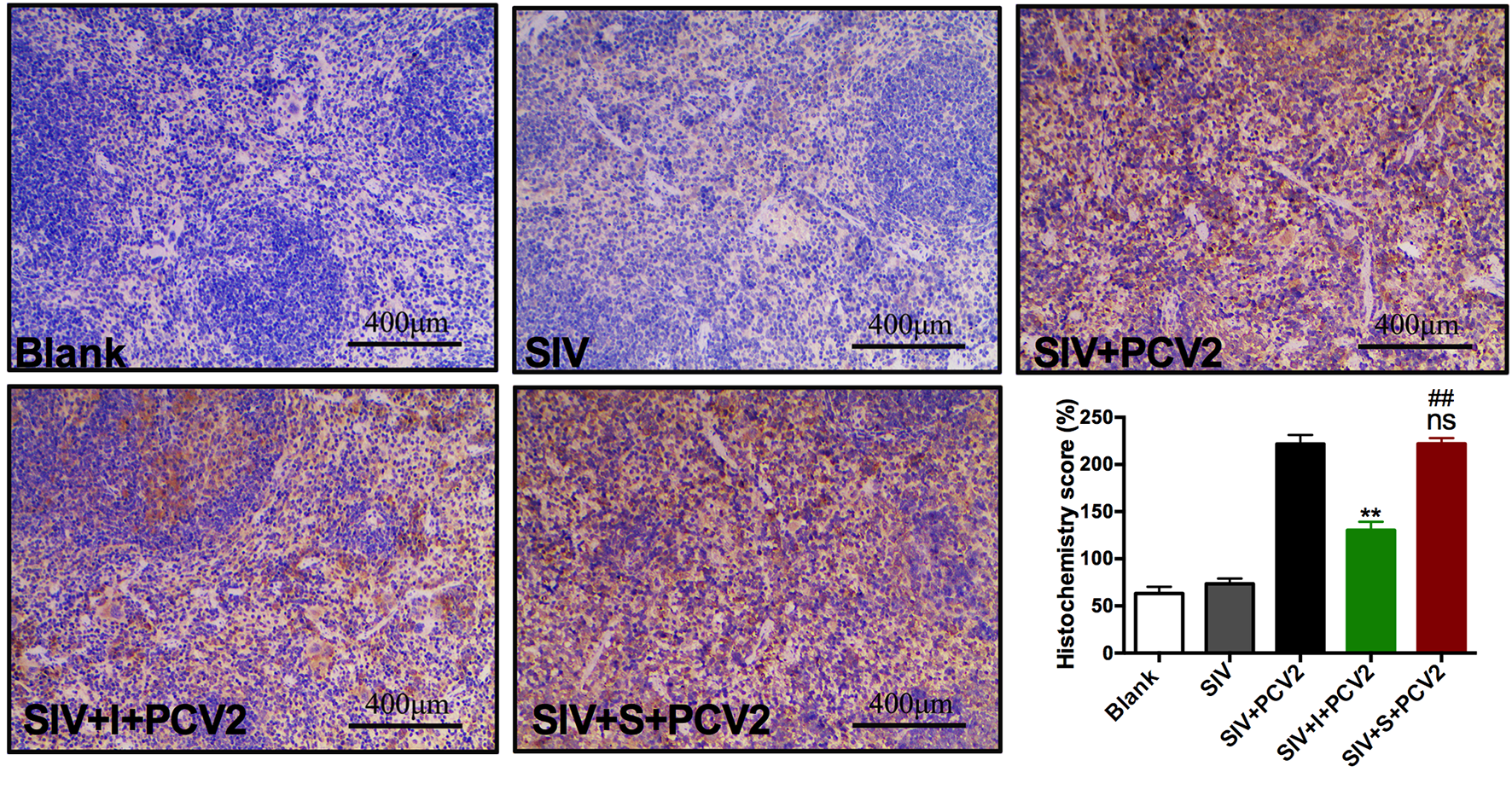

Supplement: Supplementary Figure 3 — IHC analysis for the cap expression in spleens of SIV-infected mice. The cap-specific staining intensity was showed by H-score. Compared with PCV2 group, **P < 0.01 and ns, not significant; Compared with vaccine I group, ##P < 0.01. [file Image_3.TIFF]

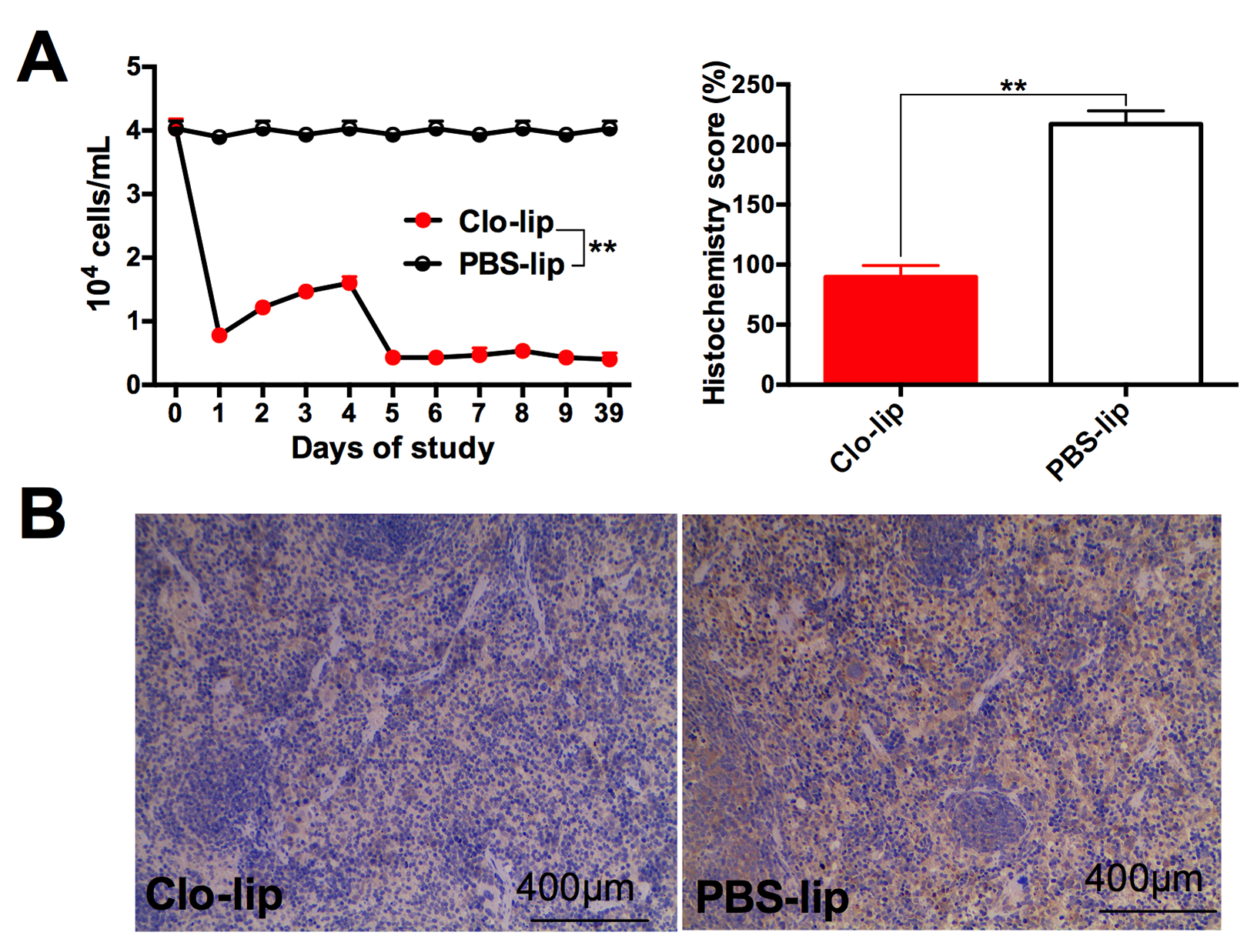

Supplement: Supplementary Figure 4 — Macrophage numbers and cap expression. (A) The number of macrophages from BAL sample was detected and calculated according to the presented time point. (B) IHC analysis for the cap expression in spleens. The cap-specific staining intensity was showed by the H-score. Data were presented as means ± SEM of mice (n ≥ 3) in each group. **P < 0.01. [file Image_4.TIFF]
